# Antibiofilm activity of *Clitoria ternatea* flowers anthocyanin fraction against biofilm-forming oral bacteria

**DOI:** 10.1093/femsle/fnaf035

**Published:** 2025-03-24

**Authors:** Allimalar Sathiaseelan, Keang Peng Song, Hock Siew Tan, Wee Sim Choo

**Affiliations:** School of Science, Monash University Malaysia, Bandar Sunway, 47500 Subang Jaya, Selangor, Malaysia; School of Science, Monash University Malaysia, Bandar Sunway, 47500 Subang Jaya, Selangor, Malaysia; School of Science, Monash University Malaysia, Bandar Sunway, 47500 Subang Jaya, Selangor, Malaysia; School of Science, Monash University Malaysia, Bandar Sunway, 47500 Subang Jaya, Selangor, Malaysia

**Keywords:** *Actinomyces viscosus*, *Aggregatibacter actinomycetemcomitans*, antiplaque, blue pea flowers, caries, *Streptococcus mutans*

## Abstract

This study investigated the antibiofilm effects of *Clitoria ternatea* flowers anthocyanin fraction (AF) on *Streptococcus mutans, Actinomyces viscosus*, and *Aggregatibacter actinomycetemcomitans*. AF was obtained using column chromatography, and liquid chromatography-mass spectrometry was employed for its characterization and identification. The crystal violet assay and scanning electron microscopy analysis revealed significant inhibition of early biofilm formation and destruction of preformed biofilms after AF treatment (0.94–15 mg ml^−1^). Antiadhesion assay on acrylic teeth demonstrated that AF effectively hampered sucrose dependent and independent attachment. Importantly, growth curve and pH drop assays showed that AF inhibited pH reduction for all bacteria tested without hindering bacterial growth. Furthermore, the tetrazolium-based cytotoxicity assay indicated no toxicity towards normal human gingival fibroblasts at 0.78–12.5 mg ml^−1^. These findings suggest *C. ternatea* anthocyanins are promising antibiofilm agents for oral biofilm control, acting during both initial formation and on mature biofilms.

## Teaser

Anthocyanins from *Clitoria ternatea* flowers have antibiofilm activity against biofilm-forming oral bacteria.

## Introduction

Biofilms, complex communities of microorganisms encased in a protective matrix, pose a significant challenge in healthcare due to their heightened antimicrobial resistance. Dental caries (tooth decay), a prime example of a biofilm-related disease, result from the complex interplay of oral bacteria. Early colonizers like *Streptococcus mutans* and *Actinomyces viscosus* initiate the process (Foster and Kolenbrander 2004, Dige et al. 2009, Fine et al. 2010). Their acid production and biofilm formation pave the way for establishing more pathogenic bacteria like *Aggregatibacter actinomycetemcomitans*, known for its persistent biofilms (Oettinger-Barak et al. 2013). Preventing the attachment and acid production of these early colonizers could be crucial in combating dental caries (Argimón and Caufield 2011).

While antiplaque agents like fluoride, triclosan, and chlorhexidine exist, their limitations, such as the potential side effects of chlorhexidine (Van Strydonck et al. 2012), motivate the search for alternative solutions for biofilm control. Secondary metabolites from plants offer a promising source of potential antimicrobial and antibiofilm compounds (Roy et al. 2018, Lahiri et al. 2019, Guimarães et al. 2021). These phyto-constituents can target and disrupt various biofilm components and formation processes.

Anthocyanins, a class of water-soluble pigments responsible for the vibrant red, purple, and blue colours in many fruits, vegetables, and flowers, are glycosylated derivatives of anthocyanidins. Pelargonidin, delphinidin, cyanidin, peonidin, petunidin, and malvidin are among the most commonly occurring anthocyanidins in plants (Choo 2019). Traditionally, anthocyanins have been used as medicine and natural food colorants (Vidana Gamage et al. 2021). The anthocyanins in *C. ternatea* flowers possess diverse biological activities, including antioxidant, antibacterial, antibiofilm, anticancer, and anti-inflammatory properties (Jeyaraj et al. 2022a). In the context of oral health, the antibiofilm potential of anthocyanins is particularly noteworthy. Studies have shown that grape marc anthocyanin extract can reduce dental caries and surface roughness in rats infected with *S. mutans* (Zagnat et al. 2017). Similarly, cranberry proanthocyanidins, structurally related to anthocyanins, have been shown to reduce *S. mutans* biofilm biomass, extracellular polysaccharides, and acidogenicity without affecting bacterial viability (Koo et al. 2010). However, the effects of *C. ternatea* anthocyanin fraction (AF) on biofilm-forming oral colonizers and its potential cytotoxicity remain unexplored.

This study investigated the antibiofilm potential of *C. ternatea* AF against *S. mutans, A. viscosus*, and *A. actinomycetemcomitans*. Additionally, the effects of bacterial adhesion, acid production, and its potential cytotoxicity on human gingival fibroblast cells (HGF-1) were investigated.

## Material and methods

### Plant material preparation and extraction of samples

Fresh *C. ternatea* flower petals (8.5–9.0° Brix) were obtained from a floriculture in Subang Jaya, Selangor, Malaysia. Ten gram of *C. ternatea* flower petals were extracted with 50% ethanol (200 ml) under continuous agitation for 3 h at 25°C. The resulting extract was vacuum filtered, concentrated with a rotary evaporator (45°C), freeze-dried (−80°C), and subsequently stored at −80°C for analysis.

### Partitioning and semipurification by column chromatography

The crude extract was partitioned and semipurified using a column chromatography method (Jeyaraj et al. 2022a). The freeze-dried extract of *C. ternatea* flowers (5 g) was dissolved in 100 ml of distilled water, and the solution was adjusted to pH 2. Liquid–liquid partitioning was performed using ethyl acetate (100 ml) to remove less polar flavonoids. The aqueous phase, enriched in anthocyanins, was collected and subjected to two additional rounds of partitioning with ethyl acetate. The resulting aqueous phase was concentrated under reduced pressure at 37°C using a rotary evaporator.

Further purification of the AF was achieved using Amberlite XAD-16 resin. The column was prepared by rinsing with 1 l of purified water, followed by activation with 500 ml of 2% (w/v) aqueous sodium hydroxide. The column was then washed with 1 l of acidified water until the pH reached 3. The concentrated AF (10 ml) was loaded onto the column and rinsed with 300 ml of acidified water (pH 3) at a flow rate of 10 ml/min to remove phenolic acids. Anthocyanins were eluted using a solution of 95:5 (v/v) methanol:acidified water (pH 2). The eluted AF was concentrated, freeze-dried, and then stored at −80°C. The total anthocyanin content (TAC) of AF was determined using the pH differential method as described by Jeyaraj et al. (2021).

### Liquid chromatography-mass spectrometry (LC-MS) characterization

Liquid chromatography-mass spectrometry (LC-MS) analysis was performed following the method from a previous study (Jeyaraj et al. 2022a). For this purpose, Agilent 1290 Infinity LC system was utilized, coupled with a 6520 Accurate-Mass Q-TOF mass spectrometer. The mass spectrometer was equipped with a dual electrospray ionization source (Agilent, Santa Clara, CA). To achieve chromatographic separation, Zorbax Eclipse XDB-C18, Narrow-Bore 2.1×150 mm, 3.5 µm column was employed, along with a guard column made of the same material. The MS detection was carried out in positive mode within a mass range of 500–2000 m/z. The elution gradients were composed of acetonitrile/methanol (1:1), formic acid (0.1:99.9, v/v) for phase A, and formic acid/water (0.1:99.9, v/v) for phase B. The elution conditions were set as follows: from 0 to 2 min, 2% A and 98% B; from 3 to 5 min, 5% A and 95% B; from 5 to 30 min, 20% A and 80% B; from 30 to 72 min, 35% A and 65% B; from 72 to 83 min, 100% A and 0% B; from 83 to 85 min, isocratic conditions with 100% A; from 87 to 90 min, 2% A and 98% B to return to the initial conditions. The flow rate was maintained at 0.5 mL min ^−1^, with an injection volume of 1 µl. Nitrogen gas was employed as the desolvation gas at 300°C with a flow rate of 60 l h ^−1^, and He gas was used as damping gas, declustering potential 40 of eV; collision energy 5 eV; collision cell entrance potential 10 eV.

### Bacterial strains and growth conditions

This study utilized *Streptococcus mutans* (ATCC 25175), *Actinomyces viscosus* (ATCC 43146), and *Aggregatibacter actinomycetemcomitans* (ATCC 33384). All strains were initially grown from glycerol stocks on brain heart infusion (BHI) agar plates.

### Assessment of minimum inhibitory concentrations (MICs)

Minimum inhibitory concentration (MIC) was determined using the broth microdilution method outlined by the Clinical and Laboratory Standards Institute (2012). Bacteria were grown overnight in BHI broth at 37°C, anaerobically, adjusted to 0.5 McFarland standard to obtain OD_600nm_ of 0.1 with 1% glucose. Serial dilutions of the AF in distilled water (1.87–60 mg ml^−1^) were prepared, and 100 µl of bacterial inoculum was added to each well. Plates were incubated anaerobically for 24 h at 37°C (Anaerogen Pak Jar, Oxoid Ltd.) and the OD at 600 nm was measured using a microtiter plate reader. Chlorhexidine was used as a positive control. The MIC was determined in triplicate using biological replicates (*n* = 3). The MIC was defined as the lowest AF concentration preventing visible bacterial growth and demonstrating a significant change in absorbance compared to the positive control (*P* ≤ .05). In cases with ≥90% inhibition, that concentration was considered the MIC.

### Assessment of minimum bactericidal concentration (MBCs)

Bacterial suspensions (100 µl) from wells without visible growth in the MIC assay were subcultured onto BHI agar plates and incubated for 24 h at 37°C under anaerobic conditions. The MBC was determined in triplicate using biological replicates (*n* = 3). The MBC was defined as the lowest concentration preventing bacterial growth on the fresh plates.

### Biofilm inhibition assay

The ability of AF to prevent bacterial biofilm formation was assessed using a modified O'Toole (2011) microplate assay. Bacteria were grown overnight in BHI broth at 37°C, adjusted to 0.5 McFarland standard, to obtain OD_600_ of 0.1 with 1% glucose. The bacterial suspension (100 µl) was added to 96-well plates along with sterile-filtered AF (0.9375–15 mg ml^−1^). Plates were incubated for 24 h at 37°C. Wells were then rinsed, fixed with 2.5% glutaraldehyde, and stained with 200 µl of a 0.1% crystal violet. Bound dye was extracted with 200 µl of 95% ethanol and absorbance was measured at 600 nm. The OD values were then converted to percentages relative to the untreated control. The negative control consisted of a bacterial suspension in broth without any treatment, while the positive control included a bacterial suspension with 0.2% chlorhexidine, a disinfectant known for its ability to eliminate oral biofilms effectively. Control wells that were not inoculated with bacteria were included to account for any background staining from the media or other components. The biofilm inhibition assay was performed with three biological replicates (*n* = 3), each biological replicate had three technical replicates. The minimum biofilm inhibitory concentration (MBIC) is initially assessed by visually identifying the lowest AF concentration that prevents visible biofilm formation. This is then confirmed quantitatively as the lowest concentration, resulting in a significant reduction in OD readings compared to the negative control, indicating a significant reduction in biofilm biomass.

### Biofilm eradication assay

The standardized inoculum (similar to the one used in the MBIC assay) was used to establish biofilms in all wells for 24 h at 37°C, anaerobically. The wells were rinsed with PBS to remove any nonadherent planktonic cells before being treated with AF (final concentration: 0.9375–15 mg ml^−1^), and incubated for another 24 h. After rinsing with distilled water, the crystal violet assay (as described in the biofilm inhibition section) was performed. The biofilm eradication assay was performed with three biological replicates (*n* = 3), each biological replicate had three technical replicates. The minimum biofilm eradication concentration (MBEC) is initially assessed by visually identifying the lowest extract concentration, resulting in a clear well (no turbidity). This is then confirmed quantitatively as the lowest AF concentration, resulting in a significant reduction in the OD reading compared to the untreated control.

### Growth curve assessment

To ensure that AF exhibited antibiofilm effects without exerting bactericidal activity at its MBIC, the effect of AF at sub-MIC on the total bacterial growth within the well was monitored over 24 h. The assay was performed with three biological replicates (*n* = 3), each biological replicate had three technical replicates.

### Acid production in the biofilm system

The effect of AF on bacterial acid production was assessed following a modified protocol from Sathiaseelan et al. (2019). BHI broth supplemented with 1% sucrose and AF (0.9375–15 mg ml^−1^) was inoculated with overnight bacterial culture and incubated for 24 h at 37°C. Initial and final pH levels of the broth were measured using a calibrated microelectrode. An untreated control (bacterial culture without the addition of AF, but with distilled water) was included for comparison. The assay was performed with three biological replicates (*n* = 3), each biological replicate had three technical replicates.

### Antiadhesion assay on artificial teeth

The effect of AF on oral bacteria's adherence to artificial teeth was assessed following a modified version of Sathiaseelan et al. (2019). One resin-based artificial tooth (VIPI-DENT plus, Madespa, Toledo, Spain) was pre-disinfected and placed in BHI medium with or without 5% sucrose. A standardized bacterial inoculum (3.5 × 10^9^ cells ml^−1^) and sub-MIC AF were added. Solvent controls (with or without sucrose) and a 0.2% chlorhexidine as the positive control were included. Incubation was under anaerobic conditions at 37°C for 4–6 h. Planktonic cells were removed, and adhered cells were detached by sonication. Adherence was quantified by measuring absorbance at 600 nm, with adherence percentage calculated by: (O.D. adhered cells/O.D. total cells) × 100. The antiadhesion assay was performed with three biological replicates (*n* = 3), each biological replicate with three technical replicates.

### Scanning electron microscopy (SEM) analysis

The antibiofilm effect of AF was visualized on glass coverslips using SEM, following the method of Sathiaseelan et al. (2017). Biofilms were grown on coverslips in 24-well microplates supplemented with AF at MBIC and MBEC concentrations (0.94–7.5 mg ml^−1^) and incubated overnight at 37°C. Biofilms were then fixed with 2.5% glutaraldehyde, rinsed with PBS, and dehydrated using a graded ethanol series. Dried biofilms were mounted on conductive tape, gold-sputtered, and examined using a Hitachi S3400N-II scanning electron microscope. To quantify the bacterial cells in the SEM images, ImageJ software (version 1.50b, National Institutes of Health, Bethesda, USA) was utilized. SEM images were taken from three independent biofilms (biological replicates, *n* = 3), with at least five images captured per biofilm at different locations on the coverslip. The images were first converted to 8-bit greyscale. A consistent threshold was applied to all images to differentiate bacterial cells from the background. The “Analyze Particles” function was used to count the individual bacterial cells in a defined area of each image (150 µm × 150 µm). The number of cell counts was log-transformed and standardized per unit area (log cells/cm^2^) of the specimen.

### Evaluation of the cytotoxic effect on cell line HGF-1

The cytotoxicity of AF was assessed using a 3-(4,5-dimethylthiazol-2-yl)-2,5-diphenyl-2H-tetrazolium bromide (MTT) assay. HGF-1 were grown in Dulbecco's Modified Eagle's Medium (DMEM) supplemented with 10% fetal bovine serum and 1% penicillin-streptomycin solution. HGF-1 (1 × 10^4^ cells ml^−1^) were seeded in a 96-well microtiter plate and incubated for 24–48 h under a 5% CO_2_ atmosphere. After incubation, cells were treated with various concentrations of AF (15–0.117 mg ml^−1^) in serum-free medium. A control well contained only cell suspension with culture medium. Following 24 h of incubation, MTT solution (5 mg ml^−1^ in PBS) was added, and plates were incubated for another 4 h at 37°C. The supernatant was removed, formazan crystals were dissolved in DMSO, and cell viability was determined by measuring the OD at 570 nm. The cytotoxicity assay was performed with three biological replicates (*n* = 3), each biological replicate had three technical replicates. Cell viability percentages were calculated relative to control cells (set at 100%). A 0.2% chlorhexidine solution served as a positive control.

### Statistical analyses

All experiments were carried out in independent triplicates. The statistical analysis was performed using SPSS version 13.0 for Windows (SPSS Inc., Chicago, IL). The results were reported as mean ± standard deviation (*n* = 3). To assess the variations between the test groups, a one-way analysis of variance test was employed. Additionally, Tukey's multiple comparison post-test was utilized to compare the means. A *P*-value less than .05 indicated a statistically significant difference.

## Results and discussion

### Characterization of anthocyanins

The vibrant blue colour of blue pea flowers is attributed to their rich anthocyanin content. LC-MS analysis of the AF identified nine major ternatins, including those from the B, C, and D classes (ternatin B1, B2, B3, B4, C1, C2, D1, D2, D3), along with several isomers, predominantly belonging to the ternatin class (Table S1). This is consistent with the flower's characteristic coloration of previous studies (Escher et al. 2020, Jeyaraj et al. 2022a, Vidana Gamage and Choo 2023). This is further supported by the absence of these pigments in white *C. ternatea* varieties (Oguis et al. 2019). Ternatins are polyacylated derivatives of delphinidin 3,3′,5′-triglucoside, characterized by multiple acyl groups, often p-coumaric acid, attached to the core anthocyanidin structure. These acyl groups not only contribute to the stable blue colour but also play a crucial role in the bioactivity of these compounds (Kazuma et al. 2003). Notably, ternatin D-type compounds and their isomers were particularly abundant. The TAC of AF was determined to be 28.19 ± 0.95 mg CGE g^−1^. This finding aligns with the results of Jeyaraj et al. (2022a), who reported a TAC of 25.9 ± 2.4 mg CGE/g, indicating successful extraction of anthocyanins from *C. ternatea* flowers.

### Minimum inhibitory concentrations and minimum bactericidal concentrations

The MIC of AF was determined as 7.5 mg ml^−1^ for both *S. mutans* and *A. viscosus. A. actinomycetemcomitans* showed a higher MIC at 15 mg ml^−1^, demonstrating lower sensitivity to the treatment (Table S2). The MBC (minimum bactericidal concentration) was 15 mg ml^−1^ for *S. mutans* and double that (30 mg ml^−1^) for *A. viscosus* and *A. actinomycetemcomitans* (Table S2). This indicates bactericidal effects at higher concentrations and bacteriostatic effects at lower concentrations.

The enhanced susceptibility of Gram-positive bacteria (*S. mutans* and *A. viscosus*) compared to the Gram-negative *A. actinomycetemcomitans* aligns with general trends in antibiotic resistance, often attributed to the protective outer membrane of Gram-negative bacteria (Breijyeh et al. 2020). This observation is also supported by previous studies demonstrating the greater efficacy of plant extracts against Gram-positive bacteria (Cisowska et al. 2011, Mahmad et al. 2018).

The observed MICs for the AF differ from those reported by Jeyaraj et al. (2022a), who investigated the antimicrobial activity of a similar fraction against *Escherichia coli, Bacillus subtilis*, and *Bacillus cereus*. This discrepancy is likely due to variations in bacterial strains, experimental conditions, and potential seasonal differences in the chemical composition of *C. ternatea* flowers.

The higher MIC and MBC values (Table S2) for *A. actinomycetemcomitans* underscore its known resistance profile, consistent with previous findings using *C. ternatea* extracts (Subianto et al. 2023) and other agents (Bakri and Douglas 2005; Oettinger-Barak et al. 2013). This bacterium's resilience may be attributed to its adaptability and ability to modulate virulence factors (Fong et al. 2003).

### Effect on biofilm formation

**Figure 1.**
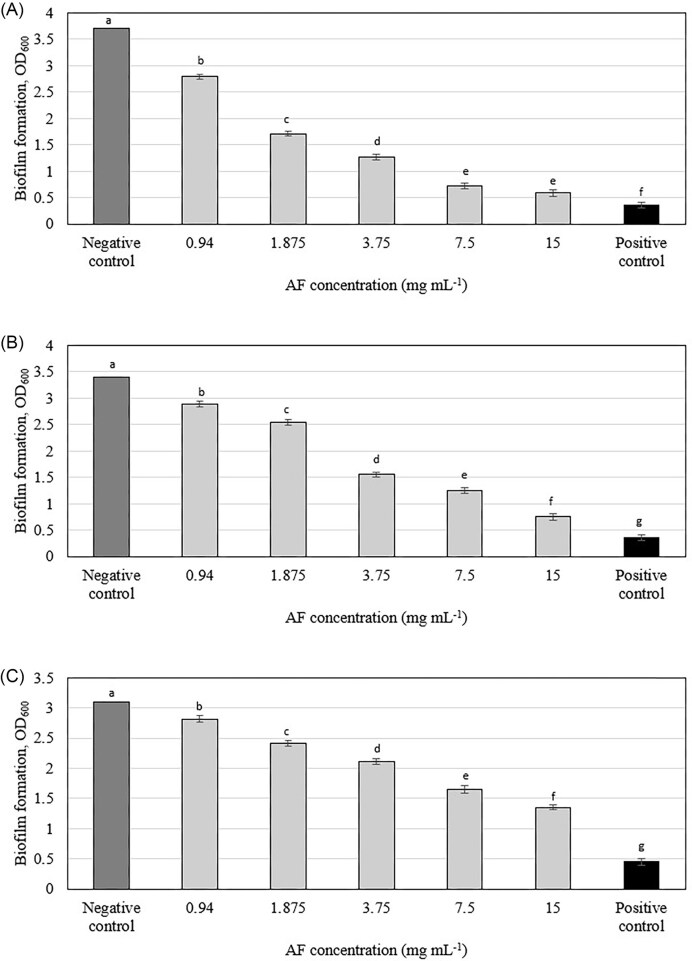
Effect of *C. ternatea* flowers AF on biofilm formation of (A) *Streptococcus mutans* (B) *Actinomyces viscosus*, and (C) *Aggregatibacter actinomycetemcomitans*. Means with different letters indicate significant (*P* < .05) differences among the treatments.

The *C. ternatea* AF demonstrated significant inhibition of biofilm formation in all three tested oral pathogens at sub-inhibitory concentrations (Fig. S1). *S. mutans* displayed the highest susceptibility, with inhibition rates ranging from 95% to 58%, followed by *A. viscosus* (93%–30%) and *A. actinomycetemcomitans* (85%–18%). The MBIC_50_ (the concentration inhibiting 50% of biofilm formation) was lowest for *S. mutans* (0.94 mg ml^−1^) and highest for *A. actinomycetemcomitans* (3.75 mg ml^−1^) (Table S2). OD measurements (Fig. 1) revealed a dose-dependent inhibition of biofilm formation for all bacterial species tested. The observed species-specific variation in susceptibility to the AF could be attributed to several factors, such as differences in cell wall composition among Gram-positive (*S. mutans* and *A. viscosus*) and Gram-negative (*A. actinomycetemcomitans*) bacteria might influence the penetration and interaction of AF with cellular targets, variations in efflux pump mechanisms, which can actively expel compounds from bacterial cells, or the presence of specific resistance genes or variations in metabolic pathways could also play a role in the differential susceptibility.

Previous studies have demonstrated the antibiofilm activity of anthocyanins from red wine, grapes, cranberries, black plum, and purple highland barley bran against various bacteria, including *S. mutans, P. aeruginosa, K. pneumonia*, and *S. enterica* (Daglia et al. 2010, Koo et al. 2010, Gopu et al. 2015, Zagnat et al. 2017, Zhang et al. 2020). Cranberries, for instance, have been well-studied for their anthocyanins' ability to reduce biofilm formation by *S. mutans* (Koo et al. 2010; Castellanos et al. 2024). Similarly, anthocyanin extracts from soybeans and walnuts have been reported to inhibit biofilm formation in *Pseudomonas* and *Klebsiella* species by reducing bacterial cell hydrophobicity and adhesion to surfaces (Enaigbe et al. 2020).

The antibiofilm activity of the *C. ternatea* AF in this study is likely due to its diverse ternatin profile, particularly the abundance of ternatin D-type compounds and isomers. These polyacylated anthocyanins, with multiple sugar moieties enhancing solubility and acyl groups contributing to stability and membrane interactions, may disrupt bacterial integrity within the biofilm. The antibiofilm activity against oral pathogens aligns with Jeyaraj et al. (2022b), who demonstrated antibiofilm activity of similar anthocyanins fraction predominately ternatin D-type, against *P. aeruginosa*.

### Effect on preformed biofilm

**Figure 2.**
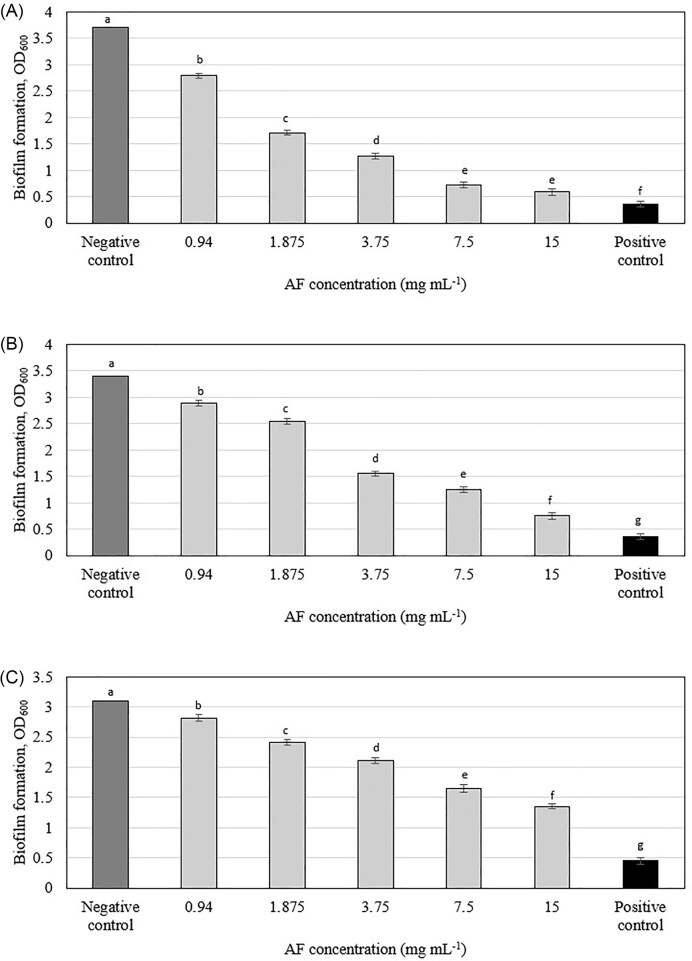
Effect of *C. ternatea* flowers AF on preformed biofilm formation of (A) *Streptococcus mutans*, (B) *Actinomyces viscosus*, and (C) *Aggregatibacter actinomycetemcomitans*. Means with different letters indicate significant (*P* < .05) differences among the treatments.

The *C. ternatea* AF demonstrated dose-dependent efficacy in eradicating pre-formed oral biofilms (Figs 2 and S2), a finding that diverges from the results of Jeyaraj et al. (2022b), who reported no significant disruption of preformed *P. aeruginosa* biofilms by the same fraction. The observed difference in AF's effect on preformed biofilms between our study (oral bacteria) and Jeyaraj et al. (2022b) (*P. aeruginosa*) might not necessarily indicate specificity to oral bacteria alone. It is possible that AF has varying efficacy against different bacterial species or that other factors such as biofilm composition, bacterial physiology, or experimental conditions contribute to this discrepancy. Future studies should investigate the effects of shorter AF exposure times on oral biofilms, assessing the impact on biofilm biomass, viability, and structural integrity. This would provide a more realistic assessment of AF's potential for clinical use. Additionally, conducting biofilm assays on artificial teeth, which more closely mimic the natural tooth surface, could offer a more clinically relevant model for evaluating AF's efficacy.

### Effect on bacterial growth

To investigate if the anti-biofilm activity of the *C. ternatea* AF was due to direct growth inhibition, bacterial growth curves were monitored over 24 h (Fig. S3). AF did not significantly affect planktonic bacterial growth, suggesting that the observed antibiofilm effect is not primarily due to bactericidal or bacteriostatic action. This is consistent with Sterniša et al. (2022), who highlighted that biofilm-targeting agents that do not directly inhibit bacterial growth are less likely to induce resistance mechanisms, potentially offering a more efficient approach to biofilm control.

### Effect on acid production in the biofilm system

The pH-drop assay revealed significant differences in acidogenic potential between untreated and AF-treated *S. mutans* biofilms. Untreated biofilms rapidly acidified the environment, reaching a pH below the critical value of 5.5, known to promote tooth demineralization, within the first 3 h, as shown in Table S3 (Selwitz et al. 2007). Conversely, AF-treated biofilms exhibited significantly suppressed acid production, maintaining a pH above 5.5 even after 24 h. This suggests that anthocyanins may not only reduce biofilm EPS content, hindering acid diffusion but also potentially inhibit enzymes involved in the glycolytic pathway of *S. mutans* (Belli and Marquis 1991), thus reducing acid production.

This finding is consistent with previous studies demonstrating the acidogenicity-reducing effects of anthocyanins from various sources against *S. mutans*. Cranberry polyphenols, including anthocyanins, disrupt *S. mutans* biofilm development and acidogenicity (Koo et al. 2010). Similarly, cranberry and blueberry extract inhibits *S. mutans* acid production, potentially through disruption of glucosyltransferases (GTFs) crucial for biofilm development (Philip et al. 2019). Grape-derived anthocyanins and polyphenols have also been shown to suppress *S. mutans* growth, acid production, and biofilm activity (Furiga et al. 2009). These collective findings underscore the potential of anthocyanins as natural anticaries agents by targeting both biofilm formation and acid production.

### Effect on bacterial attachment

**Figure 3.**
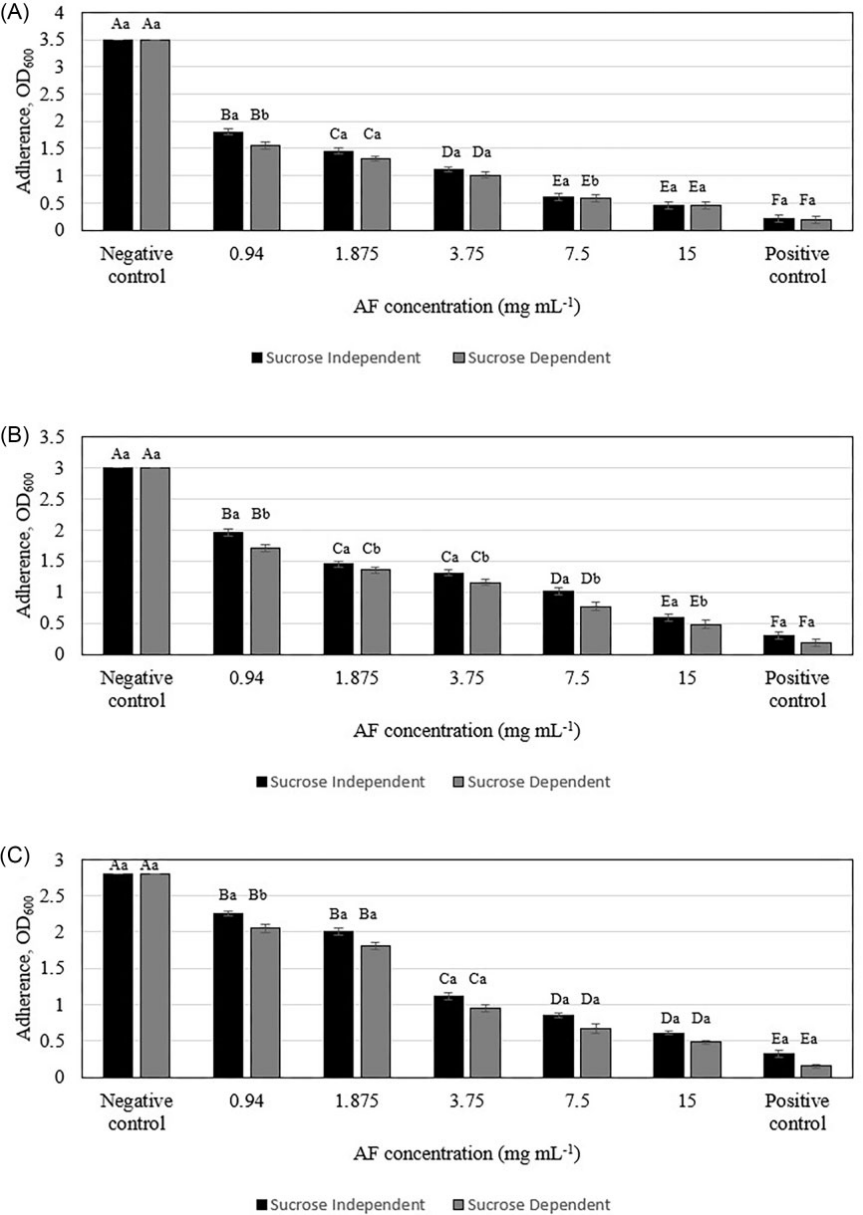
Inhibitory effect of *C. ternatea* flowers AF in sucrose-independent and sucrose-dependent adherence assays against (A) *Streptococcus mutans*, (B) *Actinomyces viscosus*, and (C) *Aggregatibacter actinomycetemcomitans*. Different letters indicate significant difference at *P* < .05 (capital letters: between different concentrations of the same attachment, lowercase letters: between different attachments of the same concentration).

*In vitro* assessment of *C. ternatea* AF using acrylic teeth as a model for natural tooth surfaces revealed significant dose-dependent inhibition of bacterial adhesion (Figs 3 and S4). This effect was observed across both sucrose-dependent and sucrose-independent attachment mechanisms, indicating a multifaceted antiadhesive action. Sucrose-dependent attachment involves the synthesis of extracellular polysaccharides (glucans) by bacterial enzymes, primarily GTFs and fructosyltransferases, which facilitate bacterial adhesion to the tooth surface (Koo et al. 2010). The observed reduction in sucrose-dependent attachment suggests that AF may interfere with these enzymes or the glucan synthesis process. Sucrose-independent attachment involves various mechanisms, including interactions between bacterial adhesins and host receptors on the tooth surface. The decrease in sucrose-independent adherence observed with AF treatment may be due to alterations in bacterial cell surface properties, such as hydrophobicity or charge, which could hinder initial attachment.

The ability of *C. ternatea* AF to inhibit early oral bacterial adhesion, a crucial step in biofilm formation that becomes increasingly irreversible over time, suggests its potential as a preventative agent against oral biofilm development. This effect is in line with previous studies demonstrating the antiadhesive properties of various plant extracts against oral pathogens. Hanafiah et al. (2018) reported a 50% reduction in *S. mutans* biofilm adhesion with 40 mg ml^−1^ of their extract, while this study found a similar reduction with a much lower concentration of AF (0.94–15 mg/ml). Similarly, Sathiaseelan et al. (2024) observed a 50% decrease in *S. mutans, A. viscosus and A. actinomycemcomitans* adhesion with 0.94–15 mg ml^−1^ of red pitahaya betacyanins fraction, which is comparable to the 50% reduction in adhesion observed in this study with AF. Importantly, this antiadhesive effect occurs at concentrations of 0.94–15 mg ml^−1^ without any significant effect on planktonic bacterial growth (Fig. 3), indicating a targeted mechanism that could be exploited for novel antiplaque strategies. These findings demonstrate the antiadhesive properties of AF against oral bacteria, and further research is needed to determine if this effect extends to other bacteria.

### SEM visualization of fixed biofilms

**Figure 4.**
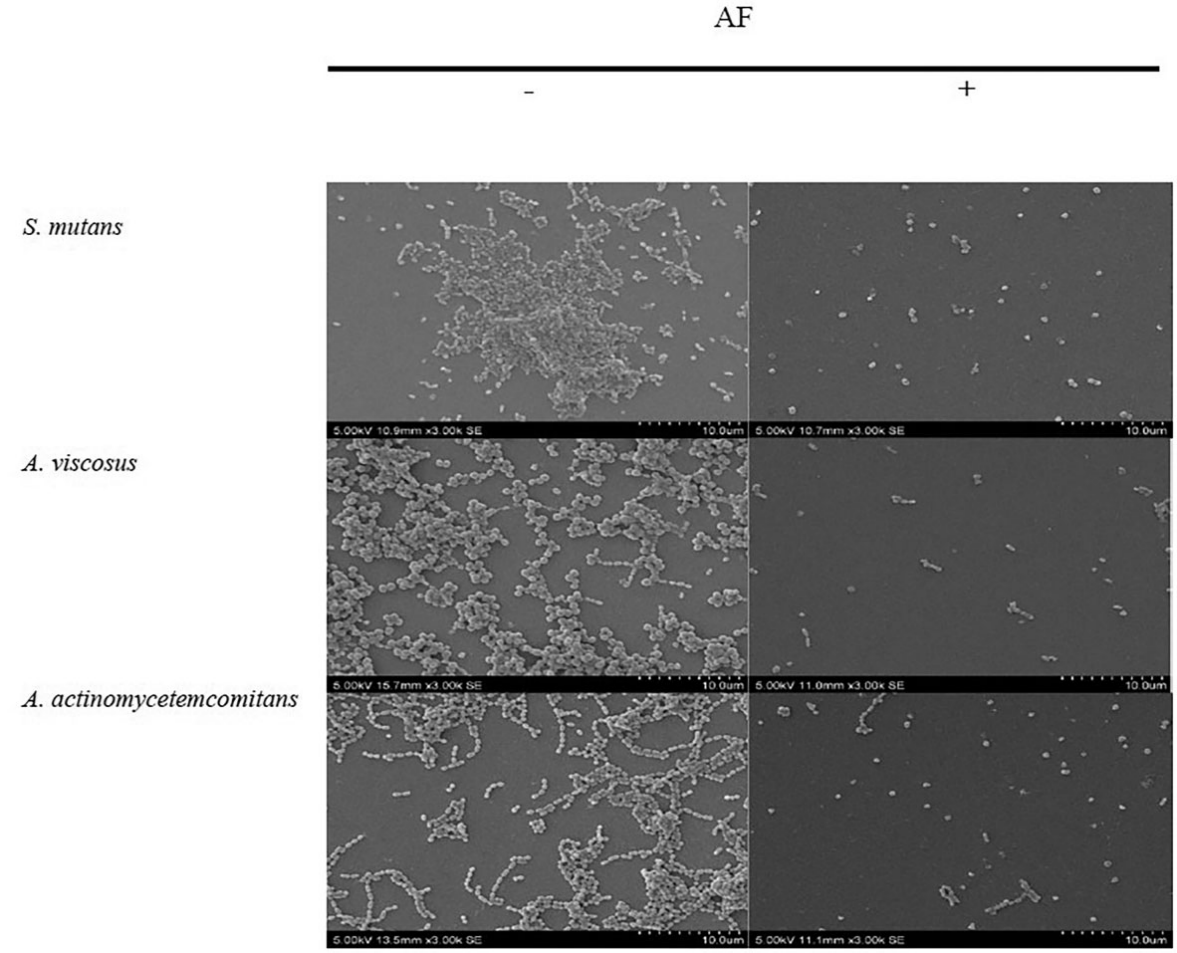
SEM images of the effect of *C. ternatea* flower AF at MBIC on biofilm inhibition at 3000× (bar, 10 µm) magnification. —indicates no treatment; + indicates with treatment.

**Figure 5.**
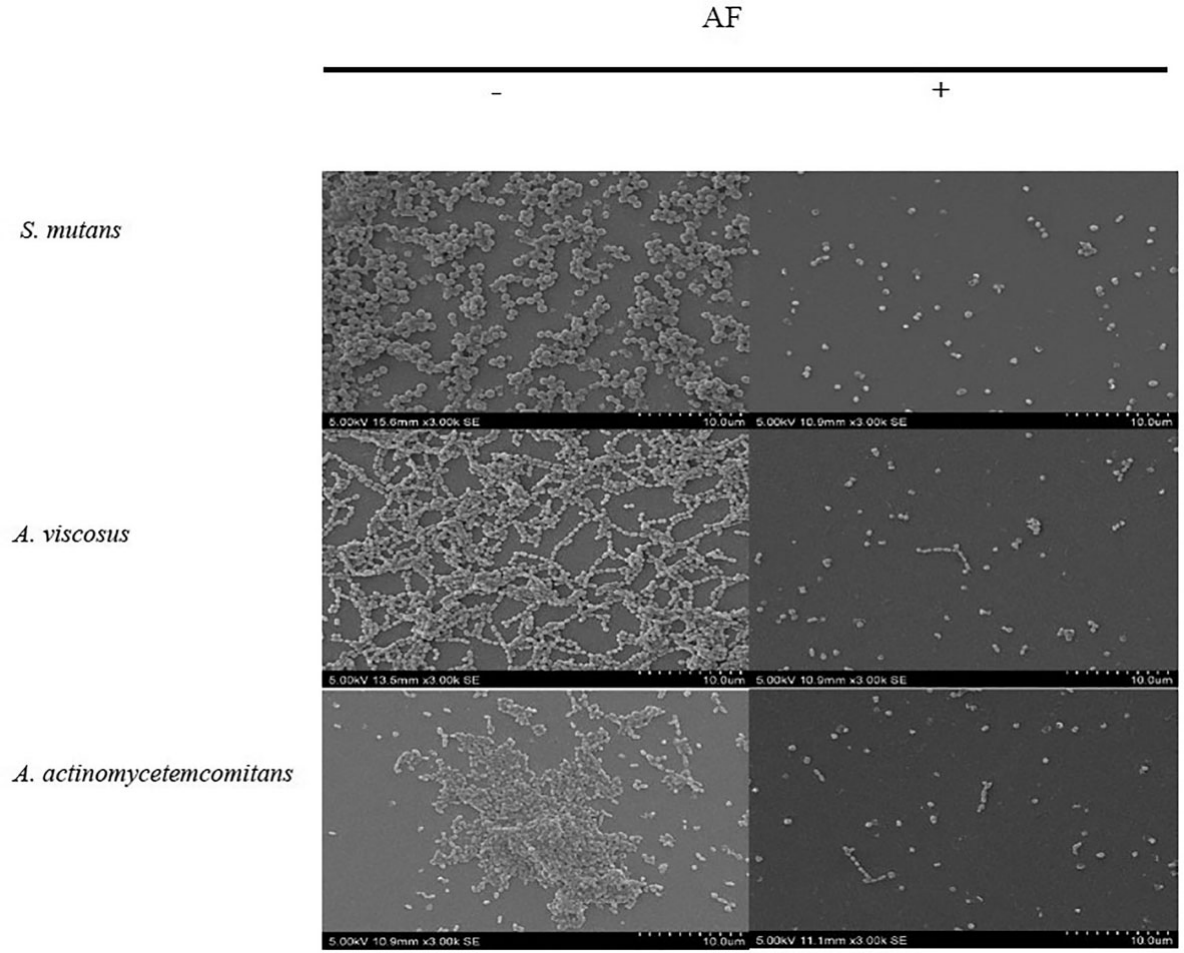
SEM images on the effect of *C. ternatea* flower AF at MBEC on biofilm destruction at 3000× (bar, 10 µm) magnification. —indicates no treatment; + indicates with treatment.

SEM of *S. mutans* biofilms revealed a marked reduction in cell density and disruption of biofilm architecture following AF at MBIC and MBEC levels (Figs 4 and 5), demonstrating a significant 1.0–1.3 log cells cm^−2^ decrease in bacterial cell density compared to the untreated control (Table S4 and S5). Control biofilms exhibited a thick, uniform matrix, while AF-treated biofilms showed progressive disruption. The treated biofilms were sparser, with smaller, less densely packed bacterial clusters. These observations were further supported by the results from crystal violet (Figs 1 and 2) and bacterial attachment assays (Fig. 3), which revealed a significant reduction in biofilm biomass and adhesion. These findings, along with previous reports of cranberry anthocyanin-induced membrane abnormalities and cytoplasmic leakage (Wu et al. 2008, Lacombe et al. 2010), suggest that AF disrupts biofilm integrity and may affect the viability of bacterial cells within the biofilm.

### Cytotoxicity assessment

**Figure 6.**
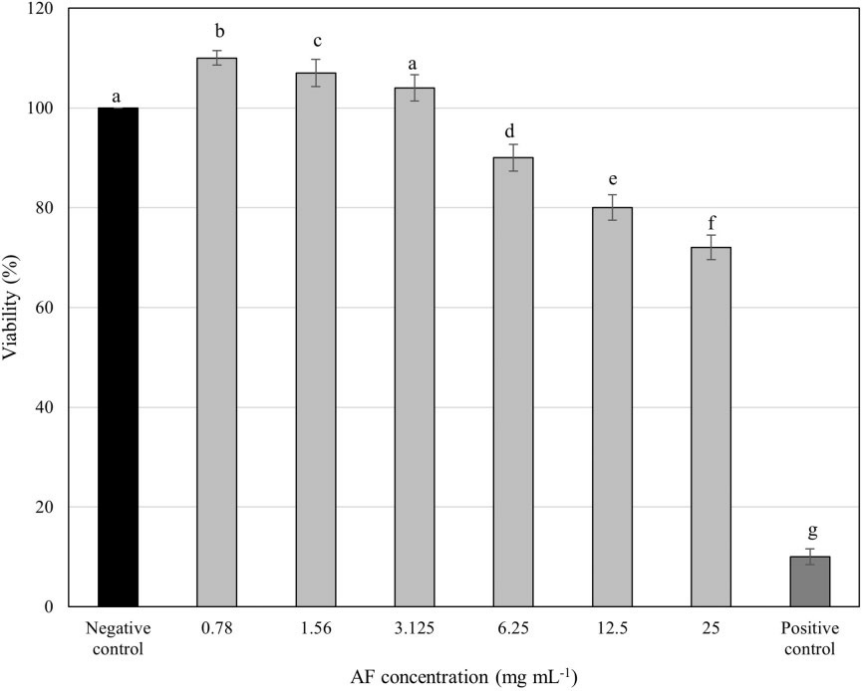
Viability of HGF-1cells treated with various concentrations of *C. ternatea* flower AF at 24 h. Cells lysed by 0.2% CHX were used as the positive control, while the untreated cells were assayed as the negative control. Different letters indicate significant difference at *P* < .05.

To assess the safety of *C. ternatea* AF for potential oral care applications, its cytotoxicity was evaluated on HGF-1 cells, the primary cells responsible for repairing oral periodontal tissues (Bartold et al. 2000). The AF exhibited no significant cytotoxic effects on HGF-1 cells across a concentration range of 0.78–12.5 mg ml^−1^ over 24 h, maintaining cell viability above the 80% threshold for healthy cultures (Fig. 6).

These findings align with previous studies demonstrating minimal toxicity of *C. ternatea* anthocyanins on normal cells, including human embryonic kidney cells (HEK-293) (Jeyaraj et al. 2022a) and human foreskin fibroblast cells (Hs27) (Neda et al. 2013). This suggests that *C. ternatea* anthocyanins exhibit a favourable safety profile for potential use in the oral cavity. Interestingly, *C. ternatea* extracts have shown cytotoxic effects against various human cancer cell lines (Shivaprakash et al. 2015), suggesting that cancer cells may be more sensitive than normal human cells.

Importantly, the MBIC and MBEC values (Table S2) for AF against *S. mutans, A. viscosus*, and *A. actinomycetemcomitans* were significantly lower than the cytotoxic threshold. This indicates that AF effectively inhibits and disrupts biofilms in a dose-dependent manner, even at concentrations well-tolerated by normal cells, highlighting its potential as a safe and effective antibiofilm agent.

## Conclusions

This study demonstrates the potent antibiofilm and anti-adherence properties of the *C. ternatea* AF against oral bacteria. Significantly, it inhibits biofilm formation and disrupts preformed biofilms without directly impacting bacterial growth. This specificity suggests a targeted antibiofilm mechanism rather than broad microbicidal activity, potentially minimizing the risk of developing bacterial resistance. Furthermore, the lack of cytotoxicity towards normal HGF-1 cells highlights its potential safety profile. These results suggest that the *C. ternatea* AF holds promise as a prospective antibiofilm agent specifically targeting oral biofilms.

## Supplementary Material

fnaf035_Supplemental_File
