# Supplementary material for: Antibiofilm activity of Clitoria ternatea flowers anthocyanin fraction against biofilm-forming oral bacteria
Source: FEMS Microbiol Lett. 2025 Mar 24;372:fnaf035. doi: 10.1093/femsle/fnaf035 (PMC11974385; doi:10.1093/femsle/fnaf035)
Supplement: fnaf035_Supplemental_File [file fnaf035_supplemental_file.docx]

**Table S1.** Characterisation of anthocyanins in *C. ternatea* flower AF via LC-MS analysis.

| Assigned compound (or isomer) | Molecular formula | Molecular ion [M+ H]^+^ | Fragment ions |
| --- | --- | --- | --- |
| 1. Ternatin C2 | C_66_H_75_O_39_ | 1491.3898 | 773.2135, 1021.2457 |
| 2. Ternatin B4 | C_60_H_65_O_34_ | 1329.3364 | 465.1190,611.1619, 788.4313, 1021.2462 |
| 3. Ternatin D3 | C_54_H_55_O_29_ | 1167.2703 | 859.1929, 1021.2451 |
| 4. Ternatin B3 | C_75_H_81_O_41_ | 1637.4276 | 1329.3352 |
| 5. Ternatin B2 | C_75_H_81_O_41_ | 1637.4267 | 1021.2456,1167.2826, 1329.3357 |
| 6. Ternatin D3 | C_54_H_55_O_29_ | 1167.2841 | 1021.2451 |
| 7. Ternatin C1 | C_60_H_65_O_34_ | 1329.3362 | 1167.2703 |
| 8. Ternatin B1 | C_90_H_97_O_48_ | 1946.5171 | 1167.2823 |
| 9. Ternatin D2 | C_69_H_71_O_36_ | 1475.3739 | 1167.2823 |
| 10. Ternatin D3 | C_54_H_55_O_29_ | 1167.2841 | 859.1929 |
| 11. Ternatin D1 | C_84_H_87_O_43_ | 1783.4643 |  |

**Table S2.** Minimum inhibitory concentration (MIC), minimum bactericidal concentration (MBC), minimum biofilm inhibitory concentration (MBIC_50_) and minimum biofilm eradication concentration (MBEC_50_) of AF against three biofilm-forming oral pathogens.

| Bacterial species | MIC (mg mL^-1^) | MBC (mg mL^-1^) | MBIC_50_ (mg mL^-1^) | | MBEC_50_ (mg mL^-1^) |
| --- | --- | --- | --- | --- | --- |
| *S. mutans* | 7.5 | 15 | 0.94 | 1.875 | |
| *A. viscosus* | 7.5 | 30 | 1.875 | 3.75 | |
| *A. actinomycetemcomitans* | 15 | 30 | 3.75 | 7.5 | |

**Table S3.** Effect of AF on bacterial acidogenicity.

| Bacterial strains | Concentration (mg mL^-1^) | pH (onset) | pH (After 24 hours of treatment) |
| --- | --- | --- | --- |
| *S. mutans* | 0  0.94  1.875  3.75  7.5  15.0 | 7.10 ± 0.05  7.07 ± 0.04  7.06 ± 0.05  7.05 ± 0.04  7.05 ± 0.03  7.06 ± 0.03 | 4.07 ± 0.04^a^  6.35 ± 0.06^b^  6.39 ± 0.02^b^  6.45 ± 0.03^b^  6.48 ± 0.04^b^  6.50 ± 0.01^c^ |
| *A. viscosus* | 0  0.94  1.875  3.75  7.5  15.0 | 7.11 ± 0.05  7.09 ± 0.04  7.08 ± 0.03  7.08 ± 0.04  7.08 ± 0.03  7.08 ± 0.04 | 4.57 ± 0.04^a^  5.89 ± 0.02^b^  6.21 ± 0.02^c^  6.29 ± 0.03^c^  6.34 ± 0.02^c^  6.41 ± 0.03^c^ |
| *A. actinomycetemcomitans* | 0  0.94  1.875  3.75  7.5  15.0 | 7.10 ± 0.05  7.07 ± 0.04  7.06 ± 0.05  7.05 ± 0.04  7.05 ± 0.03  7.06 ± 0.03 | 4.25 ± 0.04^a^  5.98 ± 0.05^b^  6.10 ± 0.02^b^  6.29 ± 0.03^b^  6.34 ± 0.06^b^  6.41 ± 0.05^b^ |

Results were presented as means ± standard deviations (*n* = 3). Different superscript letters indicate significant (p < 0.05) differences among the treatments after 24 hours in the same bacteria.

**Table S4.** Effect of *C. ternatea* AF at MBIC on the number of bacterial cells after biofilm inhibition assay on SEM images of *S. mutans*, *A. viscosus* and *A. actinomycetemcomitans*.

| Bacterial strains | Bacterial cell no. (log cells/cm^2^) | |
| --- | --- | --- |
|  | **Negative control** | **AF** |
| *S. mutans* | 7.64 ± 0.11^a^ | 6.34 ± 0.07^b^ |
| *A. viscosus* | 6.94 ± 0.13^a^ | 5.84 ± 0.50^b^ |
| *A. actinomycetemcomitans* | 6.01 ± 0.06^a^ | 4.97 ± 0.16^b^ |

^ab^ Different letters within the row indicate significant difference at p < 0.05. Data are reported as mean ± standard deviation (n = 3). CFU values represent the number of cells observed in the SEM images.

**Table S5.** Effect of *C. ternatea* AF at MBEC on the number of bacterial cells after biofilm destruction assay on SEM images of *S. mutans*, *A. viscosus* and *A. actinomycetemcomitans*.

| Bacterial strains | Bacterial cell no. (log cells/cm^2^) | |
| --- | --- | --- |
|  | **Negative control** | **AF** |
| *S. mutans* | 7.95 ± 0.11^a^ | 6.82 ± 0.24^b^ |
| *A. viscosus* | 6.14 ± 0.23^a^ | 5.10 ± 0.18^b^ |
| *A. actinomycetemcomitans* | 6.81 ± 0.06^a^ | 5.73 ± 0.11^b^ |

^ab^ Different letters within the row indicate significant difference at p < 0.05. Data are reported as mean ± standard deviation (n = 3). CFU values represent the number of cells observed in the SEM images.

f

f

f

**Figure S1.** The biofilm inhibition potential of *C. ternatea* flower AF at various concentrations against (A) *Streptococcus mutans* (B) *Actinomyces viscosus* and (C) *Aggregatibacter actinomycetemcomitans*. Means with different letters indicate significant (*p* < 0.05) differences among the treatments.

**Figure S2.** The biofilm eradication potential of *C. ternatea* flower AF at various concentrations against (A) *Streptococcus mutans,* (B) *Actinomyces viscosus* and (C) *Aggregatibacter actinomycetemcomitans*. Means with different letters indicate significant (*p* < 0.05) differences among the treatments.

**Figure S3.** Effect of *C. ternatea* flowers AF at increasing concentrations on the growth of (A) *Streptococcus mutans,* (B) *Actinomyces viscosus* and (C) *Aggregatibacter actinomycetemcomitans*.

**Figure S4.** Inhibitory effect of *C. ternatea* flower AF in sucrose-independent and sucrose-dependent assays against (A) *Streptococcus mutans* (B) *Actinomyces viscosus* and (C) *Aggregatibacter actinomycetemcomitans.* Different letters indicate significant difference at p < 0.05 (capital letters: between different concentrations of the same attachment, lowercase letters: between different attachments of the same concentration).
